# Analysis of nucleosome positioning determined by DNA helix curvature in the human genome

**DOI:** 10.1186/1471-2164-12-72

**Published:** 2011-01-27

**Authors:** Hongde Liu, Xueye Duan, Shuangxin Yu, Xiao Sun

**Affiliations:** 1 State Key Laboratory of Bioelectronics, Southeast University, Nanjing 210096, China

## Abstract

**Background:**

Nucleosome positioning has an important role in gene regulation. However, dynamic positioning *in vivo *casts doubt on the reliability of predictions based on DNA sequence characteristics. What role does sequence-dependent positioning play? In this paper, using a curvature profile model, nucleosomes are predicted in the human genome and patterns of nucleosomes near some key sites are investigated.

**Results:**

Curvature profiling revealed that in the vicinity of a transcription start site, there is also a nucleosome-free region. Near transcription factor binding sites, curvature profiling showed a trough, indicating nucleosome depletion. The trough of the curvature profile corresponds well to the high binding scores of transcription factors. Moreover, our analysis suggests that nucleosome positioning has a selective protection role. Target sites of miRNAs are occupied by nucleosomes, while single nucleotide polymorphism sites are depleted of nucleosomes.

**Conclusions:**

The results indicate that DNA sequences play an important role in nucleosome positioning, and the positioning is important not only in gene regulation, but also in genetic variation and miRNA functions.

## Background

Nucleosome positioning refers to the position of a DNA helix with respect to the histone core [1]. Positioning has important roles in gene regulation, because packing DNA into nucleosomes can limit the accessibility of the sequences [2-4]. High-resolution genome-wide nucleosome maps are now available for the genomes of yeast, worms, flies, and humans [2,5-7]. Studies of these nucleosome position datasets have revealed some interesting characteristics, especially for promoter sequences. A typical nucleosome-free region (NFR) is near the transcription start site (TSS) and is followed by a well-positioned nucleosome [4]. Low nucleosome occupancy is a significant feature of a functional transcription factor binding site (TFBS) [8].

At the same time, computational predictions using DNA sequence information have also advanced. Since the report of the nucleosome positioning code (an ~10 bp repeating pattern of dinucleotides AA-TT-TA/GC) in yeast [9], some models for predicting nucleosomes have been developed using DNA sequence properties, such as dinucleotide periodicity, and structural information of the DNA helix [5,7,10-13]. The successful predictions suggest that DNA sequences partly encode nucleosomes themselves, although some deviations are observed between the predicted and the experimentally determined positions [4,9]. On further investigation, it was realized that dynamic positioning is a general rule in cells. Dynamic remodelling of one or two nucleosomes was revealed in yeast promoters [14]. Nucleosome reorganization of a gene might result from a cell-specific change or a condition-dependent change [15,16]. For cells from the same cell line, the first nucleosome downstream of the TSS exhibits differential positioning in active and silent genes, and such nucleosome reorganization can be induced in resting T-cells [2]. Relative positioning was also found to be a general characteristic in *Caenorhabditis elegans *[6].

Such variations of nucleosome positions *in vivo *cast doubt on the reliability of predictions based on DNA sequence characteristics [4,6]. Moreover, a recent work in yeast showed that there is no genome code in nucleosome positioning; even intrinsic histone-DNA interactions are not the major determinant [17]. Also, the mechanism by which DNA sequences guide nucleosomes positions is different between *S. pombe *and *S. cerevisiae *[18]. Nucleosome organization at the 3' end of genes conforms to the principles of statistical positioning [19].

However, some factors should be noted. Firstly, the ~10-bp periodicity of dinucleotides AA-TT-TA/GC, which is identified as a positioning code in yeast, is also found in *C. elegans*, flies, and humans [9,20-24]. Secondly, strikingly similar features, including the NFR near the TSS, and the uniform spacing of internucleosomes downstream of the TSS, are observed both in the predicted data and in the experimentally determined data [5,10,11]. Low nucleosome occupancy is encoded around functional transcription factor binding sites [8,9]). Thirdly, some sequence-dependent models are suitable for predicting nucleosome positions in multiple genomes without additional information [7,11]. In addition, the chromatin remodelling complex can establish specific local chromatin structures by reading out DNA features and targeting nucleosomes to specific positions [25]. All of the above highlight the importance of sequence preferences in positioning.

In this paper, using the curvature profile, a new model based on the curvature pattern of nucleosomal DNA, nucleosomes positions were predicted for the human genome. Patterns of nucleosomes near interesting sites, including TSSs, TFBSs, single nucleotide polymorphism (SNP) sites, and target sites of miRNAs, were thoroughly investigated. The results also demonstrated the important roles of DNA sequences in determining nucleosomes. Moreover, we revealed that nucleosomes are not only functional in gene regulation, but also in genetic variation.

## Results and Discussion

### Predictions of nucleosome positions and the estimation of sequence-dependence

**Figure 1:**
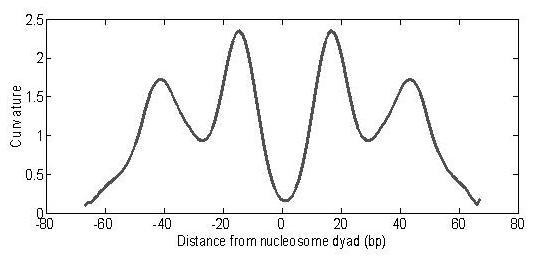
**Curvature pattern of a nucleosomal DNA helix**.

Nucleosomes positions were predicted by recognizing the curvature pattern of the core DNA helix (see methods). The reconstructed curvature pattern of a nucleosome is shown in Figure 1. To test how representative the pattern derived from the crystal structures was, 634 well-positioned (ratio of signal to noise > 100) nucleosome DNA sequences were collected from Zhao *et al*.'s experimental dataset [2]. An averaged curvature curve of the 634 sequences resembles the pattern in shape (Additional file 1: Figure s1), indicating the pattern represents a canonical curvature of a nucleosomal DNA helix.

Using the curvature profile, nucleosomes positions were predicted for human chromosome 20. A wavelet-based algorithm MSCWT [26] was employed in detecting exact dyad positions (Additional file 1: Figure s2). In the curvature profile and Kaplan *et al*.'s predictions, the averaged centre-to-centre distance of neighbour nucleosomes is ~190 bp (Additional file 1: Figure s3), close to value of 185 bp in the literatures [2,4]. For Zhao *et al*.'s experimental dataset, due to the scarcity of the coverage, this value is 245 bp, which actually indicates nucleosomal repeats rather than the average distance.

**Figure 2:**
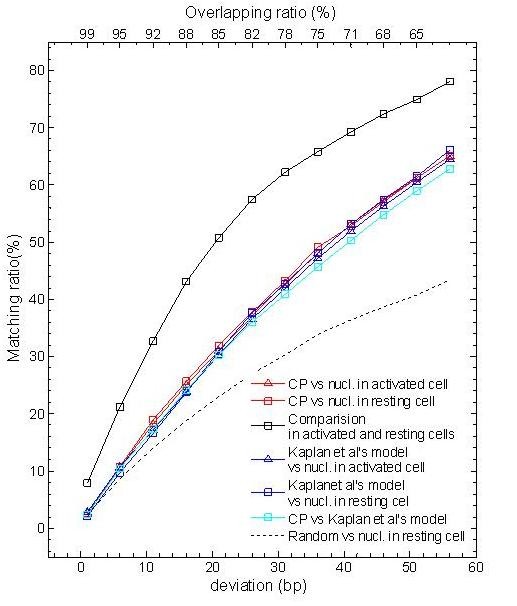
**Performance of the curvature profile for nucleosome position prediction on human chromosome 20**. Given a deviation, the matching ratio is a ratio of matched nucleosomes to all experimentally determined nucleosomes. Overlapping ratio is [2(73-deviation)+ deviation]/147, indicating the degree of overlap between the predicted and the experimentally determined nucleosomes. "CP" indicates curvature profile

A quantitative comparison between the predicted and the experimentally determined nucleosomes positions were carried out by measuring the distance between respective dyad positions (see Methods). More than 53% of the experimentally determined nucleosomes were predicted by curvature profile, with a 40-bp deviation (Figure 2). Using the experimental data as a standard, the curvature profile shows a slightly higher performance than Kaplan *et al*.'s model, especially for a low deviation (< 25 bp). Importantly, both the curvature profile and Kaplan *et al*.'s model has a much higher matching ratio for the experimental data than random positioning does. In comparison with the model nu-Score [13] on a 50k-bp sequence, the curvature profile exhibits a comparable performance (Additional file 1: Table s6). Additionally, nucleosomes have a good match in activated and resting CD4^+ ^T cells (65%, deviation > 35 bp) (Figure 2), suggesting that most nucleosomes do not change in either type of cell; only a small number of nucleosomes, such as the first nucleosome downstream of a TSS [2], exhibit different position.

**Figure 3:**
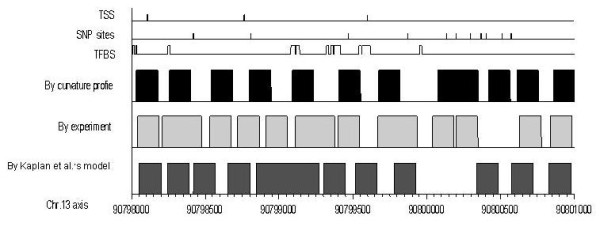
**Nucleosome predictions for a segment of human DNA sequence (chr13, 90798k-90801k bp)**. Rows from top to bottom indicate TSS, SNP sites, TFBS, nucleosomes predicted by curvature profile, experimentally determined nucleosomes [2], and nucleosomes by Kaplan *et al*.'s model [11], respectively. The filled blocks indicate nucleosomes; the original signals are shown in Additional file 1, Figure s4

Figure 3 and Figure s5 (Additional file 1) demonstrate the predictions for two arbitrarily selected DNA sequences. The positive accuracy of the curvature profile is more than 55%, with a deviation < 30 bp (Additional file 1: Table s7). Moreover, the curvature profile exactly locates most of the TSSs and TFBSs in NFRs. This indicates that the curvature profile has a good capacity for predicting nucleosome positions, especially for key functional sites.

**Figure 4:**
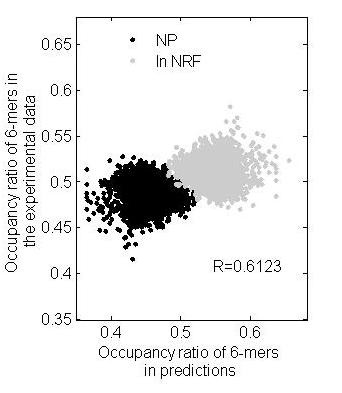
**Estimation of curvature-dependent degree of nucleosome positioning**. Black and grey dots represent the occupancy ratios and the nucleosome-free ratios of 4096 6-mer nucleotides, respectively. The estimated correlation coefficient is 0.6123; NP, nucleosome positioning; NFR, nucleosome-free region

The curvature profile is based on the curvature characteristics of nucleosomal DNA, and is therefore sequence dependent. Subsequently, the curvature-dependent degree of nucleosome positioning was estimated using the nucleosome occupancy ratio of hexanucleotides in the whole of human chromosome 20. The correlation between occupancy ratio of the predictions and the experimental data was 0.6123 (Figure 4). It should be pointed out that the curvature profile in fact reflects an indirect measurement of nucleosome position. Thus, the dependence in this study only reflects the influence of the curvature of the DNA helix on nucleosome positioning. We speculate that DNA sequences might encode a default arrangement of nucleosomes and that reorganization of nucleosomes *in vivo *is based on this default arrangement.

### Nucleosome distribution in protein-coding promoters and independent miRNA promoters

Nucleosome positioning at promoters has been extensively investigated because of its role in occluding binding sites. A typical NFR locates around a TSS, which allows the binding sites to be exposed to the pre-initiation complex (PIC) [2,5,9,11,16]. The nucleosomes flanking the NFR also provide a steric match for the complex.

**Figure 5:**
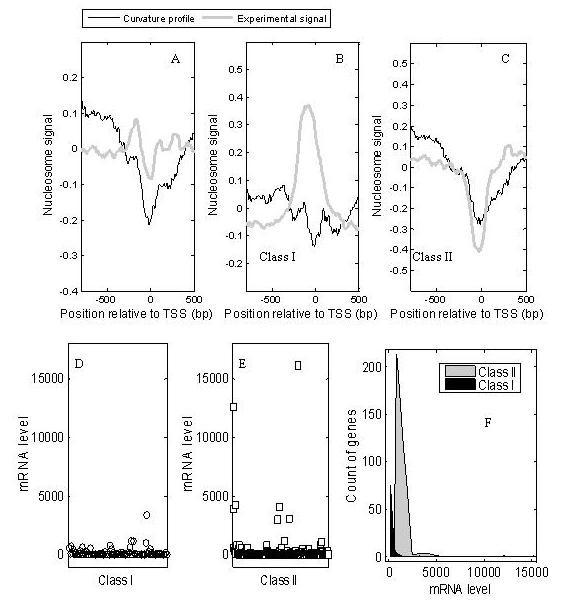
**Nucleosome organizations near transcription start sites (TSSs) in human chromosome 20**. (A), average nucleosome signals for all human chromosome 20 promoters aligned by TSS; y-axis represents an averaged scaled signal; fine line and thick grey line represent the curvature profile and the experimental data, respectively; the setting is the same for B and C; (B), nucleosome signals near class I TSSs (1080 nucleosome-occupied TSSs *in vivo*); (C), nucleosome signals near class II TSSs (2491 nucleosome-free TSSs *in vivo*); (D) and (E), the expression levels of genes in class I and class II; (F), counts of genes in according to their mRNA levels; the genes in class II have higher expression levels than those in Class I

In this study, the averaged curvature profile (fine black line in Figure 5A) results in an ~150-bp NFR around the TSS of 3571 protein-coding genes, which is consistent with previous reports [2,5,9,11,16]. The NFR is also revealed by Zhao *et al*.'s experimental data in activated CD4^+ ^T cells (grey line in Figure 5A). However, not all TSSs are nucleosome free *in vivo*. Using Zhao *et al*.'s experimental data in the range of -150 bp to 50 bp from the TSS, 3571 TSSs were divided into two classes by a k-means clustering method. This resulted in 1080 occupied TSSs (class I) and 2491 nucleosome-free TSSs (class II). Both the curvature profile and the experimental data are consistent with nucleosome depletion at class II TSSs (Figure 5C). Around the class I TSSs, no distinct positioning signal is observed in the curvature profile (Figure 5B), while a positioned nucleosome is suggested by the experiment data. This difference between the prediction and the experiment indicates that positioning is not completely determined by DNA sequences *in vivo*. The trough near the TSS in Figure 5B is slightly higher than that in Figure 5C, suggesting that a minority of class I TSSs are occupied by DNA sequence-encoded nucleosomes. As shown above, the nucleosome-free state is the default configuration, partly determined by DNA sequences at a TSS; however, *in vivo*, due to the function of the remodelling complexes, some of TSSs are occupied. This is why there are differences between the prediction and the experiment.

We computed the dinucleotide distribution near all the TSSs. Fraction of WW (W = A or T) dinucleotides decreased in a broad range near the TSS (Additional file 1: Figure s6A), Interestingly, in the range of ~100 bp upstream of the TSS, WW shows a sharp increase (Additional file 1: Figure s6A), also corresponding to an increase of poly (dA:dT) (Additional file 1: Figure s6B). We inferred that the increased poly (dA:dT) is associated with the NFR near the TSS, because the poly (dA:dT) disfavors nucleosome formation [17,18]. The results suggest that DNA sequence influences nucleosome positioning.

Taking these results together, DNA sequences partly encode a default NFR around a TSS. Due to the requirement of gene expression *in vivo*, some TSSs are occupied through chromatin remodelling, while others are still in NFRs. The positioned nucleosome at a TSS can block the binding sites of the pre-initiation complex, implying that a TSS-occupied gene should exhibit a lower expression level. This implication was verified by examining mRNA levels using gene expression data in CD4^+ ^T cells [27]. As shown in Figure 5F, the mRNA levels of TSS-occupied genes (class I) are lower than those of nucleosome-free TSS genes (class II). A similar result is observed using Zhao *et al*.'s gene expression data in activated CD4^+ ^T cells (see Additional file 1: Figure s7). The results indicate that curvature-dependent nucleosomes partly determine gene expression level.

**Figure 6:**
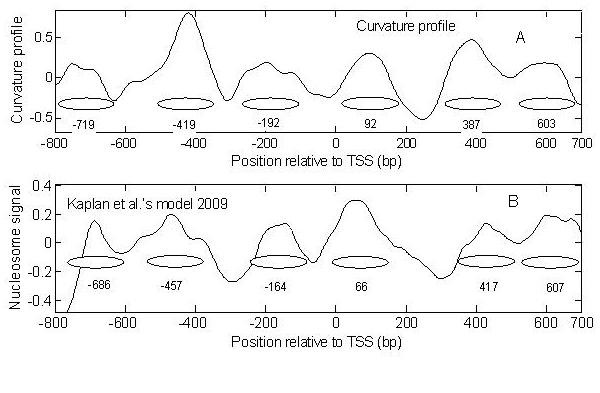
**Average nucleosome organization in the vicinity of an miRNA TSS**. (A), curvature profile; (B), Kaplan *et al*.'s model prediction [11]; ellipses indicate nucleosomes; the numbers below them indicate the dyad position relative to transcription start sites

Subsequently, nucleosome positioning at miRNA promoters was investigated. We predicted nucleosomes of miRNA promoters with the curvature profile and Kaplan *et al*.'s model [11]. Surprisingly, both models give a unanimous result (Figure 6). The mean of the deviations is less than 25 bp. Moreover, the TSSs of miRNA promoters were exactly located in NFRs, which is similar to protein-coding promoters. Two well-positioned nucleosomes flank the NFR. The results suggest that nucleosome positioning is involved in the regulation of protein-coding genes and of miRNA genes.

**Figure 7:**
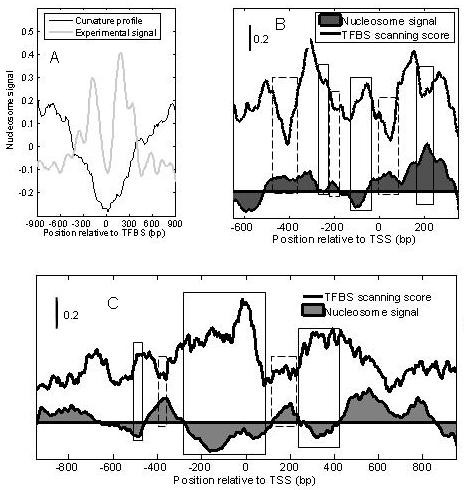
**Patterns of nucleosomes near transcription factor binding sites (TFBSs)**. (A), patterns of nucleosomes near transcription factor binding sites (TFBS); (B) and (C), the relationship between nucleosome positioning and the distribution of TFBS, (B) for 13 independent MiRNA promoters [37], (C) for eight protein-coding genes' promoters (ZBED4, ZNF378, PIK4CA, EWSR1, SMC1L2, NF2, ARFGAP3, KIAA0542) [38]; the bold black lines represent the distribution of TFBS (the average binding score profile of 64 TFs, see methods), the area with shading are averaged curvature profile, in which reference lines are cut-off lines. The vertical blocks highlight the correspondences between NFR and the distribution of TFBS

It has been reported that there is a low nucleosome level near a TFBS [8]. This feature of nucleosome organization was also observed in the curvature profile. In Figure 7A, a trough of nucleosome level is observed near TFBSs, suggesting open chromatin is indispensable to the binding of transcription factors. Due to the low resolution of the curvature profile, the trough in the curvature profile is broader than that determined by the experimental data.

As TFBSs are most frequently located in the NFRs of promoters, the NFR will correspond to the region with a high density of TFBSs (high scanning score of TFBS) in promoters [8,11]. This hypothesis was verified for both protein-coding promoters and independent miRNA promoters, by examining the relationship between the curvature profile and the average binding score profile of 64 human transcription factors (TFs) in the promoters. Eight protein-coding promoters and thirteen human independent miRNA promoters were used (see methods). As expected, the trough of the curvature profile corresponds well to the high binding scores of TFs, and regions with high levels of positioning signal (indicating nucleosome occupancy) have correspondingly low binding scores (see Figure 7). The mutually antagonistic relationship is obvious in the region of -600 bp to 400 bp for protein-coding promoters (Figure 7B), and -500 bp to 100 bp for independent miRNA promoters (Figure 7C). Outside of these regions, the relationship broke down, indicating that the regions mentioned above are important for TF binding. Despite the small number of genes used, the results indicate that a positioned nucleosome limits TF binding for both protein-coding promoters and miRNA promoters. Most importantly, it strongly suggests that the DNA sequences affect transcription, not only by providing special binding sites, but also by influencing nucleosome positioning.

Recent findings suggest that the mechanisms by which DNA sequences affect nucleosome positioning is distinct in some species [6,18,19,27]; and the choice of the periodical dinucleotides differs considerably from one organism to another [28], indicating the difficulty in finding a universal positioning code. However, it was observed that some important characteristics (such as NFRs) revealed by DNA sequence-based models are coincident with those observed *in vivo *[5,7-13]. DNA structure-related periodicity (~10-bp) is suggested in yeast, fly, worm, and human genomes [9,20-24]. These indicate that DNA sequence is one of the contributors to nucleosome positioning. The detail of how DNA sequence affects nucleosome positioning requires further investigation. One should be very carefully in using features derived from one organism to predict nucleosomes in other organisms. Recent studies showed that in RSC-depleted cells, nucleosomes move toward predicted sites [29]. Taken together, we speculate that DNA sequences partly determine a default pattern of nucleosomes positions, on which nucleosome reorganization is based. Thus, feature-based models can provide a view of nucleosome configuration determined by DNA sequences, and assist in finding certain key sites (such as TSSs) when an experimental dataset is absent.

### Genetic variation and nucleosome positioning

**Figure 8:**
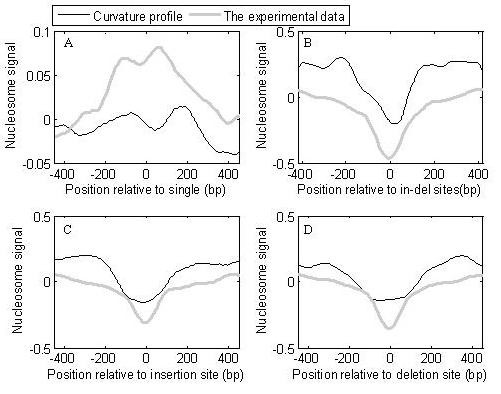
**Patterns of nucleosomes near SNP sites**. (A), single nucleotide variation (single); (B), insertions/deletions (in-del); (C), insertion SNP; (D), deletion SNP

Patterns of nucleosomes near SNP sites were investigated (see Figure 8). SNP sites are grouped into four classes, single, insertion/deletion (indels), insertion, and deletion. Near the sites of indels, insertions, and deletions, the curvature profile shows a large trough (Figure 8B-D), indicating such mutation events favor linker-DNA. Importantly, a similar result was also observed using the experimental data. Although the trough near the single SNP sites is not as low as that near the other types of SNP (Figure 8A), nucleosome depletion is obvious. In the genome of *Oryzias latipes*, genetic variation downstream of a TSS occurs with an ~200-bp periodicity, and the insertion/deletion of more than 1 bp frequently occurs in the linker-DNA region [30], which is consistent with our findings in humans. Moreover, we found that nucleosomes are also depleted near SNP sites in the dog genome (Additional file 1: Figure s8).

The above results suggest a link between nucleosome positioning and genetic variation. Nucleosome positioning has a protective role for splice sites [31]. Our findings show the opposite effect on sites depleted of nucleosomes. The sites without the nucleosomes are prone to mutations. In fact, a well-positioned nucleosome is observed at the target sites of miRNAs, protecting the key sites (see below).

### Nucleosome positioning at target sites of miRNA

**Figure 9:**
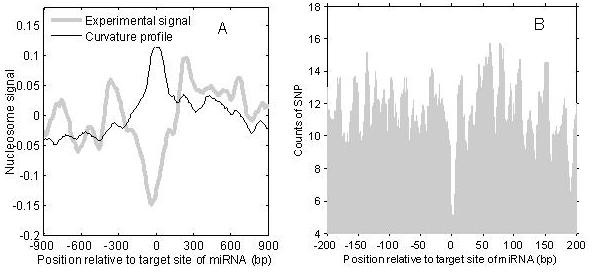
**Nucleosome positioning at miRNA target sites**. (A), patterns of nucleosomes near target sites of miRNAs; (B), distribution of SNPs in the vicinity of target sites of miRNAs in human chromosome 20

Figure 9A shows the positioning pattern of nucleosomes in the vicinity of miRNA target sites. A well-positioned nucleosome is observed at the target sites in the curvature profile. The curve of the experimental data is very similar to the curvature profile in the region far from the target sites. However, near the centre of the target sites, the experimental data gives a great valley while the curvature profile shows a peak. The similarity of two curves in the region either side of the target sites suggests that we cannot simply attribute the opposing patterns (the trough in the experimental data and the peak in curvature profile near the centre of target site) to the inaccuracy of the curvature profile. We suspect that some unknown factors prevent Zhao *et al*.'s experiment from successfully detecting the nucleosomes positioned at the target sites. Nucleosomes have a protective role for some special sites, such as splice sites [31]. miRNAs are known to be involved in post-transcriptional regulation, by binding to the 3'-UTR region of an mRNA sequence using antisense base pairing and cleaving the target mRNA or repressing its translation into protein [32]. Mutations at miRNA target sites will result in recognition errors for miRNAs. Thus, it is essential to protect the target sites of miRNAs in the nucleolus. The curvature profile's results suggest that miRNA target sites are protected by nucleosomes.

Assuming that the target sites of miRNAs are protected, the accumulation of genetic variation in target sites should be at a low level. Thus, we examined the distribution of SNPs in miRNA target sites (see Figure 9B). In range of -15 bp to 10 bp from the target sites, the SNP counts obviously decreases, indicating that the target sites are conserved in evolution. Taking into consideration the nucleosome depletion near SNP sites, it is thought that nucleosomes have a role in protecting target sites of miRNA.

As indicated above, nucleosome positioning protects some key sites, while allowing other sites to remain open. This selective protection facilitates both genome conservation and evolution. The patterns were revealed by curvature-dependent computations (the curvature profile). Therefore, genome sequences partly encode nucleosomes, and the latter allow mutations to occur at some sites and protect other special sites. Thus, nucleosome positioning has roles in genetic variation.

## Conclusions

In this study, human nucleosome positions were predicted with a curvature-dependent model; the curvature profile. The results indicate that the importance of DNA sequences in determining nucleosome positions, providing a default pattern. DNA sequence partly encodes an NFR near a TSS. *In vivo*, the TSSs of some genes are occupied by nucleosomes, and changes to nucleosome positioning will affect the genes' expression levels. In promoters, nucleosomes are depleted near TFBSs, and the distribution of TFBSs corresponds well with the NFRs. Moreover, a selective protection role of nucleosomes was revealed. SNP sites are enriched in NFRs, and miRNA target sites are associated with well-positioned nucleosomes. Our results indicate the vital role of the DNA sequence in encoding nucleosomes, and that the functions of nucleosome positioning are probably involved in further biological processes.

## Methods

### The prediction model

In our previous work [23], we found that WW (W = A or T) dinucleotides of core DNA sequences showed smaller spacing (~10.3 bp) at the two ends (~50 bp) of a nucleosome, with larger (~11.1 bp) spacing in the middle section (~47 bp). In fact, this is consistent with the cutting periodicities of core DNA [1]. Correspondingly, core DNA helices showed greater bending at the two ends, with smaller curvatures in the middle section. Using these findings, we constructed two nucleosome prediction models, the periodicity profile and the curvature profile [23]. The periodicity profile has good resolution; however, due to wavelet transformation, it is time-consuming. The curvature profile is highly efficient in computation, but overlapping peaks and significant noise decrease its resolution and hinder the recognition of nucleosomes.

To improve the resolution of the curvature profile, the curvature pattern was reconstructed in this paper. Eighteen human nucleosomal DNA sequences (146 bp) were extracted from the crystal structure dataset of the nucleosomal DNA and histone proteins (Additional file 1: Table s1). The DNA curvatures of the nucleosomal sequences were estimated using the curvature vector *C *(eq.1), which is calculated with a matrix of roll *ρ *and tilt *τ *angles (Additional file 1: Table s2), obtained for the sixteen dinucleotide steps (eq.1) [33].

(1) $$C=\nu^{0}{\left(n_{2}-n_{1}\right)}^{-1}\sum_{j=n_{1}}^{n_{2}}\left(\rho_{j}-i\tau_{j}\right)\exp\left(\frac{2\pi ij}{\nu^{0}}\right)$$

where *υ*^0 ^is the double-helix average periodicity (10.4 bp). The numbers (n_2_-n_1_) represent the integration steps. The modulus of the vector represents the deviation from B-DNA. The results generated a uniform pattern of curvature for nucleosomal sequences (see Figure 1). The pattern is called the curvature pattern of nucleosomal DNA.

Given a DNA sequence, the curvature value is calculated at each position using eq.1. The whole curvature of the sequence is called the curvature curve. The nucleosome positions can be predicted from the convolution of the curvature curve and the curvature pattern signal (Figure 1). If a segment of the curve resembles the pattern signal, the convolution will peak at the corresponding position, indicating a nucleosome. The convoluted curvature curve is called the curvature profile (Additional file 1: section 1).

### Analysis of nucleosome positioning in the human genome

Human genomic DNA sequences were retrieved from NCBI (http://www.ncbi.nlm.nih.gov/) (build 36.1), and nucleosomes were mapped using the curvature profile. To evaluate the predictions, the experimentally determined nucleosomes dataset of activated and resting CD4^+ ^T-cells was downloaded from Zhao's website (http://dir.nhlbi.nih.gov/papers/lmi/epigenomes/hgtcellnucleosomes.aspx)[2]. The dataset was used as the "standard" dataset for comparison and is called "the experimental data" in this paper. Zhao *et al*.'s dataset contains two columns for each chromosome, the first column indicates the genomic positions and the second shows scores of nucleosomes. A method based on wavelet transformation was used to detect the exact nucleosome positions from the series of scores (see below). Kaplan *et al*.'s predicted nucleosome dataset was downloaded from website (http://genie.weizmann.ac.il/software/nucleo_genomes.html) [11]. Comparisons between curvature profiles and Kaplan *et al*.'s model were carried out on human chromosome 20.

In the experimentally determined datasets, Kaplan *et al*.'s predictions, and the curvature profile, the dataset are a series of numbers. Thus, it is important to identify peaks positions to perform a quantitative comparison. Here, the maximal spectrum of continuous wavelet transformation (MSCWT) [26] (Additional file 1: section 2) was used to detect the dyad positions of nucleosomes from the raw signals of the curvature profiles and the experimentally determined nucleosomes dataset. A nucleosomal DNA was defined in a 147-bp DNA by extending 73 bp in both directions (5' and 3') from each of the dyad positions. The dataset of the dyad positions is available on our website.

The dyad positions determined by the curvature profile were compared with that in Zhao *et al*.'s experimental data by measuring the distance between respective dyad positions. Given a deviation, the amount of matched nucleosomes was counted, and a matching ratio was estimated by dividing the amount of matched nucleosomes by the total number of experimentally determined nucleosomes. The deviation varied from 1 bp to 60 bp. The comparison was performed for human chromosome 20. To explore the dynamic nucleosome positioning, the experimental datasets from both activated and resting CD4^+ ^T cells were used. Additionally, the performance of Kaplan *et al*.'s predictions was presented.

### Estimation of the sequence-dependence of nucleosome positioning

The occupancy ratio for each 6-mer nucleotide was computed by dividing the counts of nucleosome-occupancy by the counts of the hexanucleotides in human chromosome 20. The procedure was performed for both the predicted nucleosomes and the experimentally determined nucleosomes. The sequence-dependence degree was then estimated by correlating the two sets of ratios.

### Pattern of nucleosomes near special sites

More attention was paid to nucleosomes around special sites within the genome. Upstream and downstream sequences of TSSs, TFBSs [34], SNPs [35] and target sites of miRNAs [36] were extracted from UCSC (http://genome.ucsc.edu/). Information on miRNA promoters was extracted from the literature [37]. Details of these sequences are listed in Tables s3 and s4 (Additional file 1). The sequences were aligned by their special sites, and the nucleosome patterns were represented by averaged curvature profiles. To examine the effect of nucleosomes on gene expression *in vivo*, protein-coding TSSs were separated into two classes by a *k*-means clustering method using Zhao *et al*.'s experimental data [2] in a range of 150 bp upstream to 50 bp downstream of the TSS in activated CD4^+ ^T cell. One class (class I) contained TSSs that are occupied by nucleosomes; the other (class II) contained nucleosome-free TSSs. A dataset of mRNA levels [27] was used to check the effect of nucleosomes on gene expression (Additional file 1: section 3).

To test whether nucleosomes limit the binding of transcription factors, the distribution of TFBSs was computed for both protein-coding promoters (ZBED4, ZNF378, PIK4CA, EWSR1, SMC1L2, NF2, ARFGAP3, and KIAA0542) [38] and independent miRNA promoters (bold and italic in Additional file 1, Table s4 ) [37]. We scanned the binding scores of 64 human TFs (Additional file 1: Table s5) on the promoter sequences. Each TF had a unique position weight matrix (PWM); scanning with PWM on a promoter sequence resulted in a binding score profile, which indicated the potential binding regions of the TF on the sequence. The distribution of TFBSs was represented by the average binding score profile of 64 TFs on all promoters. PWMs were obtained using JASPAR [39].

An online-prediction tool for the curvature profile is provided (http://www.gri.seu.edu.cn/icons).

## Authors' contributions

HDL: model construction, data analysis, and paper preparation; SXY: web server construction; XYD: language revision and data analysis; XS: analysis of results. All authors have read and approved the final manuscript.

## Supplementary Material

Additional file 1 **Section 1 - The prediction model**. Section 2 - Detection of peak positions in curvature profiles, Zhao *et al*.'s dataset and Kaplan *et al*.'s predictions. Section 3 - Examining the expression level of TSS-occupied genes and TSS nucleosome-free genes. Table s1 - Eighteen crystal structure datasets of the DNA-histone proteins used to reconstruct the curvature characteristic. Table s2 - Values of roll ρ and tilt τ angles of sixteen dinucleotide steps. Table s3 - Details on sequences around transcription start sites (TSSs), single-nucleotide polymorphism (SNP) sites, target sites of miRNAs, start and stop codons, and boundaries of histone modifications. Table s4 - Three types of miRNAs in humans. Table s5 - Sixty-four human transcription factors used in scanning. Table s6 - Comparison of performances of the curvature profile and nu-Score. Table s7 - Prediction performance of the curvature profiles in Figure 3 and s5. Table s8 - Top 20 6-mer nucleotides that are favorable for nucleosomes and nucleosome-free regions. Figure s1 - Curvature pattern derived from 634 well-positioned nucleosome DNA sequences in the experimental dataset. Figure s2 - Identification of nucleosome dyad positions in DNA sequences from 8 k bp to 28 k bp of human chromosome 20. Figure s3 - Distribution of centre-to-centre distance of nucleosomes. Figure s4 - Predictions of nucleosomes for the segment from 90798 k bp to 90801 k bp of human chromosome 13. Figure s5 - Predictions of nucleosomes for a segment of human chromosome 17 (52269 k-52289 k bp). Figure s6 - (A) Faction distributions of WW (W = A ot T) dinucleotides and SS (S = G or C) dinucleotides near 3571 transcription start sites; (B), fraction of poly (dA) and poly (dT); (C), fraction of poly (dG) and poly (dC). Figure s7-Gene expression levels (mRNA levels) for the occupied-TSS genes (class I) and the open-TSS (class II) in activated CD4^+ ^T cells, the gene expression data is from Zhao et al's experiment (GEO accession number, GSE10437). Figure s8- Patterns of nucleosomes near SNP sites in the dog genome. Click here for file
