# Supplementary material for: Analysis of nucleosome positioning determined by DNA helix curvature in the human genome
Source: BMC Genomics. 2011 Jan 27;12:72. doi: 10.1186/1471-2164-12-72 (PMC3037905; doi:10.1186/1471-2164-12-72)
Supplement: Additional file 1 — Section 1 - The prediction model. Section 2 - Detection of peak positions in curvature profiles, Zhao et al.'s dataset and Kaplan et al.'s predictions. Section 3 - Examining the expression level of TSS-occupied genes and TSS nucleosome-free genes. Table s1 - Eighteen crystal structure datasets of the DNA-histone proteins used to reconstruct the curvature characteristic. Table s2 - Values of roll ρ and tilt τ angles of sixteen dinucleotide steps. Table s3 - Details on sequences around transcription start sites (TSSs), single-nucleotide polymorphism (SNP) sites, target sites of miRNAs, start and stop codons, and boundaries of histone modifications. Table s4 - Three types of miRNAs in humans. Table s5 - Sixty-four human transcription factors used in scanning. Table s6 - Comparison of performances of the curvature profile and nu-Score. Table s7 - Prediction performance of the curvature profiles in Figure 3 and s5. Table s8 - Top 20 6-mer nucleotides that are favorable for nucleosomes and nucleosome-free regions. Figure s1 - Curvature pattern derived from 634 well-positioned nucleosome DNA sequences in the experimental dataset. Figure s2 - Identification of nucleosome dyad positions in DNA sequences from 8 k bp to 28 k bp of human chromosome 20. Figure s3 - Distribution of centre-to-centre distance of nucleosomes. Figure s4 - Predictions of nucleosomes for the segment from 90798 k bp to 90801 k bp of human chromosome 13. Figure s5 - Predictions of nucleosomes for a segment of human chromosome 17 (52269 k-52289 k bp). Figure s6 - (A) Faction distributions of WW (W = A ot T) dinucleotides and SS (S = G or C) dinucleotides near 3571 transcription start sites; (B), fraction of poly (dA) and poly (dT); (C), fraction of poly (dG) and poly (dC). Figure s7-Gene expression levels (mRNA levels) for the occupied-TSS genes (class I) and the open-TSS (class II) in activated CD4+ T cells, the gene expression data is from Zhao et al's experiment (GEO accession number, GSE10437). Figure [file 1471-2164-12-72-S1.DOC]

# Additional file 1

**Supplementary materials**

**Section 1**

**The prediction model**

In our previous work [23], it was found that WW (W=A or T) dinucleotides of core DNA sequences were spaced at smaller intervals (≈10.3 bp) at the two ends of the nucleosome (each is 50 bp), with larger (≈11.1 bp) spacing in the middle section (47 bp). This periodicity pattern suggests that the two ends have a large curvature and the middle region has a small curvature. Subsequently, the curvature characteristic was validated with the crystal structure dataset of nucleosomes. Using the two characteristics of core DNA, namely the periodicity pattern and the curvature pattern, we constructed two nucleosome predictions models. The predictions are achieved by recognizing the pattern signal from the corresponding profile. For the model based on curvature pattern, nucleosome prediction includes four steps (see scheme 1):

1 The curvature value for a given DNA sequence is calculated using eq.1 [33]. The whole curvature of the sequence is called the curvature curve;

$C=\nu^{0}\left(n_{2}-n_{1}\right)^{-1}\sum_{j=n_{1}}^{n_{2}} \left(\rho_{j}-i\tau_{j}\right)\exp\left(\frac{2\pi ij}{\nu^{0}}\right)$ eq.1

Where $\nu^{0}$ is the double-helix average periodicity (10.4 bp). The numbers ($n_{2}-n_{1}$) represent the integration steps.

2 Carry out convolution of the curvature and the curvature pattern signal. The convolution signal is called the curvature profile. If a segment of the curve resembles the pattern signal, the convolution will give a peak at the corresponding position, indicating a nucleosome.

Given vectors *u* and *v*, with a length of *m* and *n*, respectively, the convolution of *u* and *v* is represented with vector *w*, the *k*th element of *w* is calculated with eq. 2.

$w\left(k\right)=\sum_{j=1}^{k} u\left(j\right)\cdot v\left(k-j\right)$, $k\in\left[1,\begin{matrix} \end{matrix}m+n-1\right]$ eq.2.

3 Find the positions of the peaks of the curvature profile and predict nucleosomes.

Seq: acgtacggtatgcgt……

Eq.1

Curvature curve

The curvature pattern signal

Convolution

**Scheme 1. Illustration of prediction procedure using the curvature pattern**

Matlab codes of curvature profile

% Main function

function [Conv_P]=curvature_profile(seq)

% comput the curvature profile of DNA sequence!

Ori_s=DNA_curvature(seq);% Oringal Curvature of DNA signal;

load patter_nu.mat; % load pattern of core DNA

Conv_P=wkeep(conv(Ori_s,P),length(Ori_s));

%perform converlution of pattern and DNA Curvature

end

% sub function

function sigma=DNA_curvature(seq)

step=10;

l_My_s=length(seq)-step-1;

v0=10.4;

c_u=sqrt(-1);% complex unit i

C(1)=0;sigma(1)=0;

sig(1)=0;

for i=1:l_My_s

s=seq(i:(i+step));

temp_C=0;

for j=[1:(step-1)]

dinu=s(j:j+1);

curve=curve_DNA_modi(dinu);

roll=curve(1);tilt=curve(2);

d=(roll-c_u*tilt)*exp(2*pi*c_u*j/v0);

temp_C=temp_C+d;

end

% comput curvature

C(i)=(v0/step)*temp_C;

sigma(i)=C(i)*conj(C(i));

end

end

function curve=curve_DNA(dinu)

% compute the curvature of dinucleotides

switch lower(dinu)

case 'aa'

curve=[-0.09 0];

case 'at'

curve=[0.16 0];

case 'ta'

curve=[-0.12 0];

case 'ca'

curve=[-0.04 -0.03];

case 'gt'

curve=[0.12 -0.01];

case 'ct'

curve=[0.04 0.03];

case 'ga'

curve=[-0.02 -0.03];

case 'cg'

curve=[-0.07 0];

case 'gc'

curve=[0.10 0];

case 'gg'

curve=[0.01 -0.02];

case 'tg'

curve=[-0.04 0.03];

case 'ac'

curve=[0.12 0.01];

case 'ag'

curve=[0.04 -0.03];

case 'tc'

curve=[-0.02 0.03];

case 'cc'

curve=[0.01 0.02];

case 'tt'

curve=[-0.09 0];

end

end

**Section 2**

**Detection of peak positions in curvature profiles, Zhao et al.’s dataset and Kaplan et al.’s prediction**

In both the experimentally determined dataset and the curvature profile, the data is a series of numbers. Thus, the exact peak position of the dyad axis is required to perform a quantitative comparison. In this paper, a signal processing technique, maximal spectrum of continuous wavelet transform (MSCWT) [26], was employed to detect peaks positions.

For a time series *f*(*t*), its continuous wavelet transform (CWT) is expressed in Eq.2.

$Wf\left(a,b\right)=\frac{1}{\sqrt{a}}\int_{-\infty}^{+\infty} f(t)\psi^{*}\left(\frac{t-b}{a}\right)dt$ Eq.2

Where *a* and *b* are scale and alternation parameters, respectively; and *Wf*(*t*) is the CWT result.

MSCWT is obtained by detecting the maximal module of CWT (Eq.3).

MSCWT(*t*) =max (|*Wf*(*t*)|) Eq.3

The peak position in MSCWT is the same as that in original signal, and the peak becomes sharp. In detecting the peak position, the mother wavelet is a Mexican hat function. Scale range [a1­:a2] is important in detecting peaks.

Detecting peak positions with MSCWT includes four steps:

Step 1, perform CWT on the signal (curvature profile, Zhao et al.’s dataset and Kaplan et al.’s prediction) in a scale range with eq.2. For the curvature profile, Zhao et al.’s experimental dataset and Kaplan et al.’s predictions, the proper scale ranges are [30:32], [2:6], and [30:32], respectively.

Step 2, detect and record the CWT maximum at every translation (position); that is, obtain the MSCWT of the signal.

Step 3, Set zero as the cutoff.

Step 4, find those peaks greater than the cutoff, and establish peaks positions.

Figure s2 show examples of identifying nucleosome dyad positions. The dyad positions is available on our website ([www.gri.seu.edu.cn/icons](http://www.gri.seu.edu.cn/icons)).

**Section 3**

**Examining the expression level of TSS-occupied genes and TSS nucleosome-free genes**

To examine the effect of nucleosomes on gene expression in vivo, a dataset of mRNA levels [26] was used. 3571 protein-coding TSSs were separated into two classes by a *k*-means clustering method using Zhao et al’s experimental data [2] in a range of 150 bp upstream and 50 bp downstream of TSS in activated CD4^+^ T cell. Class I contains 1080 TSSs that are occupied by nucleosomes; Class II contains 2491 nucleosome-free TSSs.

Zhao et al’s dataset of nucleosomes positions is detected in CD4^+^ T cells, and our analysis is only for chromosome 20; therefore, we extracted the expression data of genes on chromosome 20 in CD4^+^ T cells. This resulted in a dataset of mRNA levels of 552 genes. Secondly, we examined TSS positions and the ranges of genes. If a TSS was located in the range of a gene, we considered the TSS is the gene’s start site. Among 552 genes, 89 genes had nucleosome-positioned TSSs (Class I); 216 genes had nucleosome-free TSSs (Class II). Finally, the gene expression levels of two classes were investigated.

**Table s1** Eighteen crystal structure datasets of the DNA-histone proteins used to reconstruct the curvature characteristic

| 1M1A; 1P34; 1P3A; 1P3B; 1P3F; 1P3G; 1P3I; 1P3K; 1P3L; 1P3M; 1P3O; 1P3P; 1S32; 1U35; 1ZLA; 2CV5; 2F8N; 2NQB |
| --- |

**Table s2** Values of roll ρ and tilt τ angles of sixteen dinucleotide steps

| dinucleotide | ρ | τ | dinucleotide | ρ | τ |
| --- | --- | --- | --- | --- | --- |
| T->A | 0.16 | 0 | C->A | 0.12 | 0.01 |
| T->T | -0.09 | 0 | C->T | -0.02 | 0.03 |
| T->G | -0.12 | -0.01 | C->G | 0.10 | 0.00 |
| T->C | 0.04 | 0.03 | C->C | 0.01 | 0.02 |
| A->A | -0.09 | 0.00 | G->A | 0.04 | -0.03 |
| A->T | -0.12 | 0.00 | G->T | -0.04 | 0.03 |
| A->G | -0.02 | -0.03 | G->G | 0.01 | -0.02 |
| A->C | -0.04 | -0.03 | G->C | -0.07 | 0.00 |

**Table s3** Details on sequences around transcription start sites (TSSs), single-nucleotide polymorphism (SNP) sites, target sites of miRNAs, start and stop codons and boundaries of histone modifications

| Special sites | | Upstream (bp) | Downstream (bp) | Number | Location | description |
| --- | --- | --- | --- | --- | --- | --- |
| TSS | Protein-coding promoters | -750 | 500 | 3571 | chr. 20 | SwitchGear TSS, based on experimental evidence |
|  | miRNA promoters | -700 | 250 | 117 | except chr. 18 and chr. y | ref. (Ozsolak et al, 2008) [37] |
| SNP sites | | -1000 | 1000 | 11307 | chr. 20 | dbSNP build 129, ref. (Sherry et al. 2001) [31] |
| miRNA target sites | | -1000 | 1000 | 1420 | chr. 20 | predicted by TargetScanS (Lewis BP, Burge CB and Bartel DP. Cell 2005, 120(1):15-20) |

**Table s4** Three types of miRNA in human [37]

| Intron miRNA; Using host promoter (51) | hsa-mir-548b  hsa-mir-550-2  hsa-mir-553  hsa-mir-554  hsa-mir-559  hsa-mir-561  hsa-mir-566  hsa-mir-571  hsa-mir-578  hsa-mir-580  hsa-mir-589  hsa-mir-590  hsa-mir-609 | hsa-mir-616  hsa-mir-618  hsa-mir-619  hsa-mir-624  hsa-mir-627  hsa-mir-629  hsa-mir-636  hsa-mir-637  hsa-mir-641  hsa-mir-642  hsa-mir-643  hsa-let-7g  hsa-mir-103-1 | hsa-mir-140  hsa-mir-148b  hsa-mir-149  hsa-mir-152  hsa-mir-16-2  hsa-mir-185  hsa-mir-186  hsa-mir-191  hsa-mir-22  hsa-mir-25  hsa-mir-26b  hsa-mir-301  hsa-mir-326 | hsa-mir-330  hsa-mir-378  hsa-mir-423  hsa-mir-449  hsa-mir-574  hsa-mir-584  hsa-mir-615  hsa-mir-647  hsa-mir-657  hsa-mir-661  hsa-mir-7-1  hsa-mir-611 |
| --- | --- | --- | --- | --- |
| Intron miRNA With independent promoter (23) | hsa-mir-548c  hsa-mir-550-1  hsa-mir-604  hsa-mir-634  hsa-mir-635  hsa-mir-639 | hsa-let-7c  hsa-let-7e  hsa-mir-125b-2  hsa-mir-128b  hsa-mir-149  hsa-mir-153-1 | hsa-mir-20a  hsa-mir-30c-1  hsa-mir-339  hsa-mir-33b  hsa-mir-340  hsa-mir-450-1 | hsa-mir-564  hsa-mir-632  hsa-mir-658  hsa-mir-9-1  hsa-mir-98 |
| Intergenic miRNA(43) | hsa-mir-200b  hsa-mir-92b  hsa-mir-607  hsa-mir-612  hsa-mir-100  hsa-mir-196a-2 hsa-mir-18b  hsa-mir-505  hsa-mir-594  hsa-mir-320  hsa-mir-30b  hsa-let-7f-1  hsa-mir-222 | hsa-let-7i  hsa-mir-193b  hsa-mir-484  hsa-mir-195  hsa-mir-365-2  hsa-mir-10a  hsa-mir-21  hsa-mir-371  hsa-mir-10b hsa-mir-563  hsa-mir-138-1  hsa-mir-572  hsa-mir-9-2 | hsa-mir-146a  hsa-mir-219-1  hsa-mir-30c-2  hsa-mir-148a | ***hsa-mir-138-2***  ***hsa-mir-565***  ***hsa-mir-130b***  ***hsa-mir-345***  ***hsa-mir-374***  ***hsa-mir-648***  ***hsa-mir-101-1***  ***hsa-mir-135b***  ***hsa-mir-146b***  ***hsa-mir-210***  ***hsa-mir-129-2***  ***hsa-mir-30a***  ***hsa-mir-200c*** |

**Table s5** 64 human transcription factors used in scanning

| MIZF; NFYA; ESR1; NR3C1; HNF4A; NF-kappaB; TBP; Cebpa; REST; BRAC1; TFAP2A; E2F1; ELK1; GABPA; ELK4; SPI1; SPIB; ETS1; FOXF2 ; FOXD1 ; FOXC1 ; FOXL1 ; FOXI1 ; SOX9; SRY; PBX1; NKX3-1; Pdx1; LHX3; TLX1-NFIC; MEF2A; SRF; NR2F1; PPARG-RXRA; PPARG; RORA_1; RORA_2; RXRA-VDR; NR1H2-RXRA; TP53; Pax6; REL; NFKB1; RELA; STAT1; TEAD; IRF1; IRF2; MZF1_1-4; MZF1_5-13; RREB1; SP1; YY1; ZNF354; GATA2; GATA3; NHLH1; Myf; TAL1-TCF3; MAX; MYC-MAX; USF1; CREB1; NFIL3; HLF |
| --- |

**Table s6** Comparison of performances of curvature profile and nu-Score [13] on a 50k bp sequence

|  | Deviations (bp) | 25 | 30 | 35 | 40 |
| --- | --- | --- | --- | --- | --- |
| Sensitivity | Curvature profile | 0.4832 | 0.5959 | 0.6908 | 0.7682 |
|  | nu-Score | 0.4984 | 0.6026 | 0.6910 | 0.7434 |
| Specificity | Curvature profile | 0.4968 | 0.5957 | 0.7428 | 0.7821 |
|  | nu-Score | 0.4786 | 0.5944 | 0.7080 | 0.7899 |

**Table s7** Prediction performance of curvature profile in Fig.3 and Fig.s5; the predictions are compared with the experimentally determined nucleosomes [2], with a deviation of 30 bp.. The performance of Kaplan et al.’s model (http://genie.weizmann.ac.il/software/nucleo_Prediction.html, version 3.0) is also presented [11].

|  |  | TP | FP | FN | Positive accuracy (%) | Sensitivity (%) |
| --- | --- | --- | --- | --- | --- | --- |
| The first segment (chr. 17: 52269000-52289000 bp) | Curvature profile | 39 | 21 | 10 | 55.71 | 79.59 |
|  | Kaplan et al.’s model | 40 | 36 | 8 | 52.63 | 83.33 |
| The second segment (chr.13: 90798000-90801000 bp) | Curvature profile | 7 | 5 | 1 | 58.33 | 87.50 |
|  | Kaplan et al.’s model | 5 | 8 | 1 | 38.46 | 83.33 |

**Table s8** Top 20 6-mer nucleotides that are favorable for nucleosomes and nucleosome-free regions in predictions and in experimental data in vivo; NP, nucleosome positioning, NFR, nucleosome-free region

| In predictions | | *In vivo* (CD4^+^ T cell) | |
| --- | --- | --- | --- |
| NP | NFR | NP | NFR |
| CGTCCG  CGCGGG  CCGGCG  CGGCGC  TCGAAT  CGCCGC  CCGCGA  CGGCCG  CCGCCG  GCGCCG  CGAGCG  CGCGGC  GCCGCC  CGCGAA  CGGTCG  CGCGCG  CCCGCG  AGCGCG  CGGCGG  GTCCGG | AACAAC  AAACTA  ACAACA  AACTAC  CAACAA  AAAAAC  ACAAAA  AAAACC  AACAAA  ACATAC  ACACAA  ATACCA  TATACG  ACACAC  TCAACA  ACGACC  TACGAC  ACGCAA  AAACAA  ACTACC | TACGCG  ATCGCG  CGATCG  ACGCGT  GCGCGA  GGCGCG  CGAGTA  TCGCGC  GCGGAT  TTCGCG  CGCGGT  CGGATC  CGCGAT  CGCGCG  CGCGCC  CGCGTG  CGAGAC  CACGCC  CAGGCG  TCGCAC | GACTCG  TCGCAT  CGAATG  GAACGG  AGCCGT  CGTACG  CCGAAC  CGTTGT  ATCGTA  AGCGTC  GTCGAT  ATCCGT  TCGATG  CGGCGT  CGTACA  CGAACG  CGATGA  GCATCG  GCGAGT  GATTCG |

**Figure s1** Curvature pattern derived from 634 well-positioned nucleosome DNA sequences in Zhao *et al*.’s experiment dataset [2], “well-positioned” means ratio of signal to noise > 100.

(A) Averaged curvature of 634 well-positioned nucleosomes DNA sequences;

(B) Fraction distribution of WW (W=A or T) dinucleotides at each position of centre-aligned 634 nucleosomes sequences. Shown is the result with a 3-bp moving average.

(C) Same as (B), but for SS (S=G or C) dinucleotides

A

B

C

**Figure s2** Identification of nucleosome dyad position, shown is for DNA sequence from 8k bp to 28k bp of human chromosome 20

Subplots (A), the curvature profile; (B), nucleosome score in activated CD4^+^ T cell; (C) nucleosome score in resting CD4^+^ T cell; (D) Kaplan et al.’s predictions

A

B

C

D

**Figure s3** Distribution of centre-to-centre distance of nucleosome in Zhao *et al*.’s experimental data [2], curvature profile and Kaplan et al predictions [11]

**Figure s4** Predictions of nucleosomes for the segment from 90798k bp to 90801k bp of human chromosome 13. The filled blocks indicate nucleosomes; the solid lines show original signals of models (curvature profile and Kaplan et al. 2009 [11]), and the experiment. The top row shows the curvature profile; the middle row shows the signal detected by the experiment [2]. The bottom row shows the signal by Kaplan et al.’s model

Curvature profile

Experimental data

Chr. 13 axis

Kaplan et al.’s model

**Figure s5** Predictions of nucleosomes for a segment of human chromosome 17 (52269k-52289k bp). SNP sites, TFBS, nucleosomes by curvature profile, the experimental data (Zhao *et al.*, Cell, 2008) [2] and by Kaplan *et al.*’s model [11] are shown. The filled blocks indicate nucleosomes

SNP sites

By Kaplan et al.’s model

TFBS

By curvature profile

By the experiment

Chr.17 axis

**Figure s6** (A) Faction distributions of WW (W=A ot T) dinucleotides and SS (S=G or C) dinucleotides near 3571 transcription start sites; (B), fraction of poly (dA) and poly (dT); (C), fraction of poly (dG) and poly (dC)

A

B

C

## Figure s7 Gene expression levels (mRNA levels) for the occupied-TSS genes (class I) and the open-TSS (class II) in activated CD4^+^ T cells, the gene expression data is from Zhao et al’s experiment (GEO accession number, [GSE10437](http://www.ncbi.nlm.nih.gov/geo/query/acc.cgi?acc=GSE10437)).

**Figure s8** Patterns of nucleosomes near SNP sites in the dog genome
